# HypoxiaDB: a database of hypoxia-regulated proteins

**DOI:** 10.1093/database/bat074

**Published:** 2013-10-30

**Authors:** Pankaj Khurana, Ragumani Sugadev, Jaspreet Jain, Shashi Bala Singh

**Affiliations:** Bioinformatics Group, Defence Institute of Physiology and Allied Sciences (DIPAS), Defence R&D Organization, Lucknow Road, Timarpur, New Delhi-110054, India

## Abstract

There has been intense interest in the cellular response to hypoxia, and a large number of differentially expressed proteins have been identified through various high-throughput experiments. These valuable data are scattered, and there have been no systematic attempts to document the various proteins regulated by hypoxia. Compilation, curation and annotation of these data are important in deciphering their role in hypoxia and hypoxia-related disorders. Therefore, we have compiled HypoxiaDB, a database of hypoxia-regulated proteins. It is a comprehensive, manually-curated, non-redundant catalog of proteins whose expressions are shown experimentally to be altered at different levels and durations of hypoxia. The database currently contains 72 000 manually curated entries taken on 3500 proteins extracted from 73 peer-reviewed publications selected from PubMed. HypoxiaDB is distinctive from other generalized databases: (i) it compiles tissue-specific protein expression changes under different levels and duration of hypoxia. Also, it provides manually curated literature references to support the inclusion of the protein in the database and establish its association with hypoxia. (ii) For each protein, HypoxiaDB integrates data on gene ontology, KEGG (Kyoto Encyclopedia of Genes and Genomes) pathway, protein–protein interactions, protein family (Pfam), OMIM (Online Mendelian Inheritance in Man), PDB (Protein Data Bank) structures and homology to other sequenced genomes. (iii) It also provides pre-compiled information on hypoxia-proteins, which otherwise requires tedious computational analysis. This includes information like chromosomal location, identifiers like Entrez, HGNC, Unigene, Uniprot, Ensembl, Vega, GI numbers and Genbank accession numbers associated with the protein. These are further cross-linked to respective public databases augmenting HypoxiaDB to the external repositories. (iv) In addition, HypoxiaDB provides an online sequence-similarity search tool for users to compare their protein sequences with HypoxiaDB protein database. We hope that HypoxiaDB will enrich our knowledge about hypoxia-related biology and eventually will lead to the development of novel hypothesis and advancements in diagnostic and therapeutic activities. HypoxiaDB is freely accessible for academic and non-profit users via http://www.hypoxiadb.com.

Database URL: http://www.hypoxiadb.com

## Background

Hypoxia is a pathophysiological condition and refers to an abnormally low partial pressure of oxygen in atmosphere, low content of oxygen dissolved in per liter of blood or less percentage saturation of hemoglobin with oxygen, either found singly or in combination (1, 2). Different levels and duration of hypoxia cause varying adverse effects from time to time. It may occur in diseased conditions (like cancers, malignancies, etc) or may be induced by environmental factors. In malignancies, the blood supply is restricted in tumor tissue region, which leads to tumor hypoxia (3). Almost 140 million people in the world (representing ∼2% of the world’s human population) reside in high altitudes (8000 ft above sea level) and are continuously exposed to hypoxia (4). A number of studies have proven that these people are genetically better adapted to hypoxic stress (5–8). People are also exposed to hypoxia while they climb to high altitude (whole body hypoxia) or while breathing mixture of gases with low oxygen content (tissue hypoxia). Hypoxia in high altitude may lead to potentially fatal complications like high-altitude pulmonary edema (9) and high-altitude cerebral edema (10). Hypoxia is also a serious consequence of preterm birth in neonates (11). According to World Health Report 2004, almost 22.46% of deaths worldwide occurred because of hypoxia either directly or indirectly due to ischemia, chronic obstructive pulmonary disease, cancer, etc (12–14). Many research efforts have been made to identify the acquired and inherited risk factors, which is very much evident by the increase in the number of hypoxia-related conferences and symposium held worldwide periodically (15–19). However, it is still difficult to predict accurately the susceptibility and adaptability to hypoxia.

In humans, hypoxia is a multivariate disorder and is influenced by factors like varying oxygen concentrations, time duration of hypoxic exposure, altitude, physical stress and environmental and genetic factors (20). Thus, it has captured the interest of many of the research groups, and over the past decades, many experimental strategies and large-scale efforts have been undertaken for the studies of hypoxia under varying percentages of oxygen. At the molecular level, hypoxia-inducible factor-1 (HIF-1) and other members of the HIF family of transcriptional activators have provided insight into the molecular mechanisms of oxygen homeostasis. The HIF family members are critical for physiological adaptations to chronic hypoxia, which include erythropoiesis, vascularization, metabolic reprogramming and ventilatory acclimatization (21). Global gene/protein expression studies for hypoxia are widely used to identify the regulation of various genes and proteins, and a large amount of global molecular data for hypoxia has been published and accumulated over time. The reduction in the cost of the genome-wide and proteome-wide association studies has further aided the process, and massive amount of hypoxia-related data has been thrown unregulated in the public domain. To date, no database has been developed to collate the information present for the hypoxia-regulated proteins and no specific database has been dedicated entirely to hypoxia and hypoxia-related disorders. Although some of the generalized databases such as AmiGO (22), GeneCards (23), ArrayExpress (24), are present and are highly referred to by the researchers working in the field of hypoxia, these tools and databases show limited results. Also, they do not provide the detailed information such as the level of hypoxia, organ/tissue specificity, correlation of a particular protein with hypoxia, along with the other details. Thus, we have aimed to compile the high-throughput studies done for hypoxia under varying hypoxic conditions and tissue specificity so as to give the account of most of the genes/proteins that are affected during hypoxia. In this article, we aim to efficiently integrate and analyze most of the global studies published for hypoxia-regulated proteins. We collected the data for the human proteins regulated by hypoxia by intensive literature search and have manually curated all the data associated with hypoxia from various publications. For each protein, we have made useful annotations, which include correlation of proteins with hypoxia, level of regulation along with the fold change, tissues in which the genes/proteins are expressed, map location of the proteins, Gene Ontology (GO) terms and descriptions, Protein family (Pfam) information, Kyoto Encyclopedia of Genes and Genomes (KEGG) pathway information, protein–protein interactions (PPI) from Human Protein Reference Database (HPRD), Online Mendelian Inheritance in Man (OMIM) information, protein structure information from Protein Data Bank (PDB), along with the other external link IDs such as Unigene, Entrez, HUGO Gene Nomenclature Committee (HGNC), Ensembl, International Protein Index (IPI), HomoloGene, GI and Genbank accession numbers. As this is the first database for hypoxia-regulated proteins, HypoxiaDB seeks to be a useful resource for the research community involved in hypoxia and hypoxia-related research.

## Construction and content

### Data collection, curation and integration

Research articles pertaining to hypoxia-regulated gene/proteins were collected from PubMed using appropriate keywords such as hypoxia, ischemia, anoxia and *Homo sapiens*. Only those papers that report changes in expression pattern of gene/proteins were selected. They were scrutinized for relevant information. Further, Gene Expression Omnibus (GEO) database (25) was explored to identify articles that report high-throughput experiments related to hypoxia. Of these, only human studies were considered. Thus, among 500 peer-reviewed publications that were screened, 73 were selected for entering the data in the database. We organized these data by manually converting different identifiers in publications to the unique National Center for Biotechnology Information (NCBI) Entrez/Gene ID. Different articles have used different experimental techniques to prove the correlation of the proteins with hypoxia; thus, the experiments were classified into three categories, namely, genomic, transcriptomic and proteomic evidence. Thus, for each PubMed ID, the corresponding evidential experiment is listed as genomic/transcriptomic/proteomic evidence. The published reports give a list of significantly differentially regulated proteins. We have compiled the fold-change values for each protein as stated in the publication.

Protein GI numbers were extracted either from the reference papers or from BioDBnet (26). Genbank protein accessions were retrieved using E-utility applications (27). The *R* Bioconductor package org.Hs.eg.db (28) was used to convert Genbank protein accessions to Entrez ID. The HGNC ID, HPRD ID, Vega ID and Ensembl ID were extracted from ‘gene_info’ file downloaded from NCBI ftp site (ftp://ftp.ncbi.nlm.nih.gov/gene/DATA/GENE_INFO/) (29). The Pfam ID, IPI ID, Uniprot ID, Unigene ID, OMIM ID, KEGG ID, GO ID and list of all the Genbank protein accessions were parsed using the *R* package org.Hs.eg.db (28). GO annotations were extracted from ‘gene2go’ file downloaded from NCBI ftp (ftp://ftp.ncbi.nlm.nih.gov/gene/DATA/) (29). KEGG pathway information was parsed using the *R* packages org.Hs.eg.db and KEGG.db (28). In addition, intensive manual curation was done to make sure that the pathways are linked correctly. The protein family information was extracted using the *R* packages org.Hs.eg.db and Pfam.db (28). As HPRD is one of the biggest human PPI databases, we extracted the PPI information from HPRD (30). Entrez utilities (27) were used to retrieve the protein FASTA sequences. The HomoloGene database (31) enlists the homology of a protein/gene of one species with that of the other species. The HomoloGene ID was extracted using BioDBnet, and the other important HomoloGene information was parsed from ‘Homogene.xml’ file, which was downloaded from (ftp://ftp.ncbi.nih.gov/pub/HomoloGene/current/) (29).

### Data architecture and web interface

**Figure 1.**
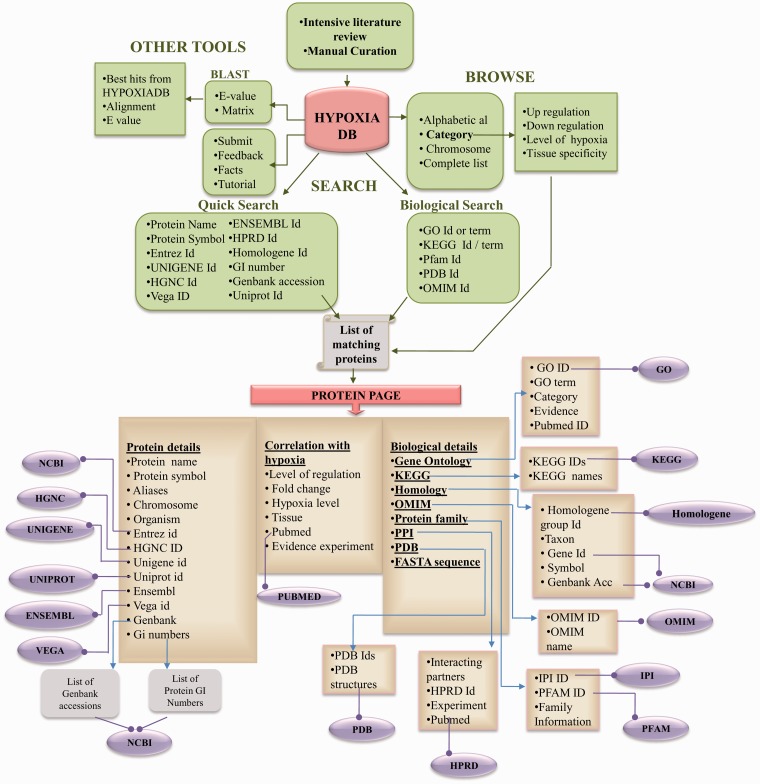
A detailed schematic architecture of HypoxiaDB database.

HypoxiaDB is built on Apache HTTP server 2.2, with CGI-Perl and Perl scripts at the back-end and the HTML, Javascript and CSS at the front-end. Apache, CGI-Perl and Perl are preferred, as these are open-source software and are platform independent. The overall layout of the database is shown in Figure 1.

## RESULTS

### User interface

HypoxiaDB provides simple and user-friendly interface to ‘browse’ and ‘search’ data. In addition, ‘other applications’ are provided.

### 1) Browse option

The browse option provides an interface to retrieve data without entering keywords. To help the users browse data conveniently, HypoxiaDB provides three different methods for browsing the hypoxia-regulated proteins:

- Browse by alphabetical order: Each alphabet links to a table that lists protein symbols and protein names starting with the respective alphabet. Each protein symbol is then linked to the corresponding protein page, which contains detailed information about the protein and its expression changes in hypoxia. The details in the protein page are discussed further. The user can also browse through the complete alphabetical list of proteins.
- Browse by category: This option allows the user to browse through the list of proteins according to the categories listed below:

- List of upregulated and downregulated proteins: Using this option, the user can retrieve a list of proteins that are up/downregulated during hypoxia.
- List of up/downregulated proteins in different levels of hypoxia: This option is useful if the user wants to browse the list of proteins that have been reported to change under specific hypoxia conditions.
- List of up/downregulated protein in a particular tissue: This option enlists the proteins up/downregulated in hypoxia in different tissues. The results can be advantageous for the user interested in tissue-specific studies.

The browse result is in the form of a table containing protein symbol, up/downregulation, fold change, percentage hypoxia, tissue, PubMed ID and type of study (genomic/transcriptomic/proteomic). The protein symbol links to the detailed protein page and the PubMed links to the specific research publication.

iii Browse by chromosomal location: The user can click on the human chromosome number to display the proteins on each chromosome and then browse them individually. Users can also browse the complete list of proteins arranged according to the chromosomal location.

### 2) Search option

Data can be searched at two levels in HypoxiaDB: (i) Quick search and (ii) search based on biological information.

#### (i) Quick search

Quick search can be done using any of the following protein identifiers, namely, protein name, protein symbol, Gene/Entrez ID, Uniprot ID, HGNC ID, Vega ID, Ensembl ID, HPRD ID, HomoloGene ID, GI number, Genbank accession and Unigene ID. Among these options, protein name and protein symbol search for similar matches, whereas the others search for exact matches. The result is a list of matching entities, which are further linked to corresponding detailed protein pages.

#### (ii) Search based on biological information

In this search option, user can give the following protein identifiers as the query term: GO ID or term, KEGG pathway ID or term, Pfam ID, PDB ID or OMIM ID.

### GO ID or term

The program searches for matches containing the query term in the GO field. For example, the query ‘GO:000’ will list all the GO IDs containing ‘GO:000’. The result is retrieved in the form of a table having the following fields, viz, Gene/Entrez ID, GO ID, GO term, category and evidence (PubMed). Each Entrez ID is linked to the respective protein page; GO ID is cross-linked to GO database; evidence is linked to the GO evidence code; and PubMed ID is linked to the article from which the evidence of the GO term was taken.

### KEGG pathway ID or term

This program searches the query term in the KEGG pathway field. The result is retrieved in the form of a table having Entrez ID, KEGG ID and KEGG term. The Entrez ID is linked to the detailed protein page, and the KEGG ID is hyperlinked to KEGG pathway database.

### Pfam ID

The query result is obtained in the form of a table having the fields Entrez ID, IPI ID, Pfam ID and protein family. The Entrez ID is linked to the detailed protein page, IPI ID is linked to the IPI database and Pfam ID is linked to the Pfam database.

### PDB ID

The program searches the query term for exact match in the PDB field. The result is a list of Entrez IDs of proteins, which take the same fold as the query PDB. The Entrez ID is linked to the detailed protein page.

### OMIM ID

The program searches for exact match in the OMIM field. The result is a list of Entrez IDs of proteins, which have Mendelian inheritance specifications matching with the enquired OMIM ID. The Entrez ID is linked to the detailed protein page.

## Detailed protein page

The information presented on the protein page is divided into three sections:

### General information

**Figure 2.**
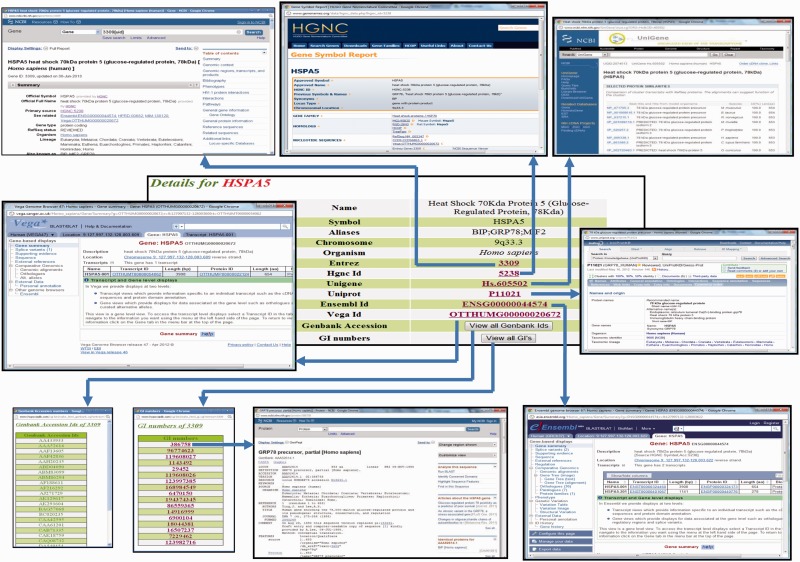
Screenshot showing the detailed protein page of HypoxiaDB. Screenshot showing the portion of the protein page, which enlists the details of the protein and cross-links HypoxiaDB to various other external databases.

The general information present on the protein page includes protein name, protein symbol, aliases, chromosome and organism name (Figure 2). The protein identifiers like Entrez ID, HGNC ID, Unigene ID, Uniprot ID, Ensembl ID, Vega ID and list of all Genbank protein accession and GI numbers are respectively cross-linked to external databases, namely, NCBI gene page, HGNC, Unigene, Uniprot, Ensembl, Vega and NCBI protein pages.

### Correlation of protein with hypoxia

**Figure 3.**
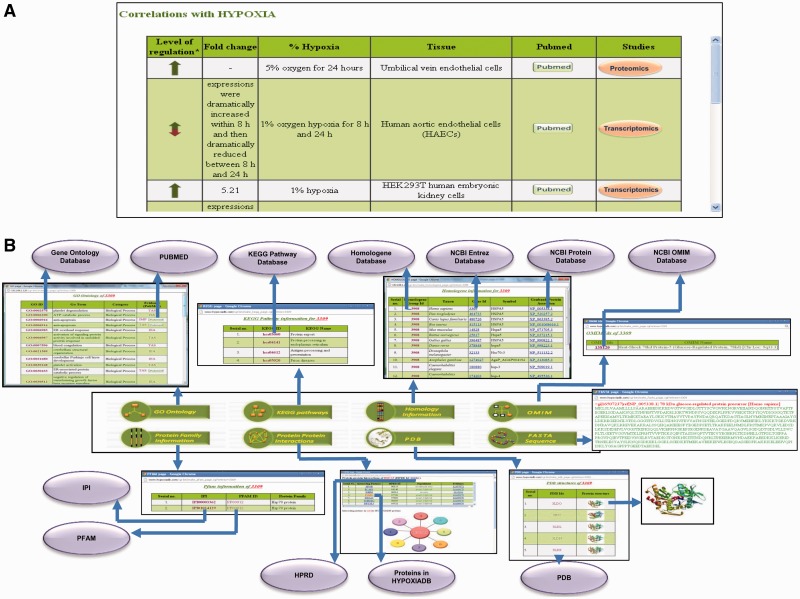
Screenshot showing the detailed protein page of HypoxiaDB. (**A**) The part of the protein page that describes the expression pattern of the protein under different levels and duration of hypoxia. The table also enlists the tissue of expression and the evidential experiment. (**B**) The last portion of the protein page provides the various biological details of each protein. The purple circles represent other public repositories cross-linked to HypoxiaDB.

HypoxiaDB is made after intensive literature search, and correlation of a particular protein with hypoxia may be demonstrated by more than one paper. Hence, this section contains tissue-specific expression pattern changes of the protein under different levels and durations of hypoxia compiled from various publications. The data are organized as level of regulation (up/down), fold change, percentage of hypoxia, tissue of expression, PubMed link to the research publication and the type of study (genomic/transcriptomic/proteomic) done to state the correlation (Figure 3A). Different tissues vary considerably in their response and sensitivity to hypoxia. Moreover, the molecular response in each tissue varies under different levels and durations of hypoxia. Henceforth, consequences are also wide-ranging. This has important implications in the management of oxygen transport and monitoring of tissue hypoxia in critically ill patients. From clinical perspective, this is significant (32–34). An analysis of various studies shows that there is a core set of proteins that are induced consistently by hypoxia and a large number of proteins that exhibit cell-type-specific induction (35). Hence, tissue-specific time-dependent expression profile of proteins is important and sought-after information for researchers in the field of hypoxia. To date, HypoxiaDB is the first study that lists tissue-specific protein expression changes under different levels and duration of hypoxia. Hence, this database serves as a rich source of information for hypoxia biologists.

### Other biological details

#### GO

This section provides information about the GO of the protein. It gives information about the GO ID, which is cross-linked to the AmiGO database, GO term, GO category and evidence (PubMed), which is further cross-linked to the research publication (Figure 3B).

#### KEGG pathway

This gives information about the KEGG IDs associated with the protein and the respective KEGG names. The KEGG ID is linked to the KEGG pathway database (36) (Figure 3B).

#### Homology information

This link provides details on the homologous proteins in other genomes. It lists the HomoloGene group ID for the protein, which is linked to HomoloGene database. Information regarding organism, Entrez ID, protein symbol and protein accession number is also present on the homology page (Figure 3B).

#### OMIM

OMIM links to the OMIM ID of the protein and OMIM name. The OMIM ID links to the OMIM database (Figure 3B).

#### Protein family information

A click on the protein family information lists the IPI ID, Pfam ID and the protein family name. The IPI ID and Pfam ID are hyperlinked to the IPI database and the Pfam database, respectively (Figure 3B).

#### PPI

This link provides the PPI information for each protein. The information is depicted in the form of a table and an interaction network figure. The figure is present only if the interacting partners are <20. It gives information about interactor’s name and its HPRD ID, interacting partners and their HPRD ID’s and evidential experiment and the respective PubMed ID. The HPRD ID links to the HPRD database. Interacting partners that are not present in HypoxiaDB are linked to NCBI. If the interacting partner is present in HypoxiaDB, it is highlighted in red and hyperlinked to the respective detailed protein page. This helps to identify proteins that are interacting in hypoxia conditions and may help in deciphering new hypoxia-linked pathways (Figure 3B).

#### FASTA sequence

The link provides the sequence of the protein in FASTA format (Figure 3B).

### 3) Other applications

The other applications available for the users at HypoixaDB include the following:

#### (i) BLASTP

A customized BLAST tool has been made available that searches user-provided query against the sequences available in the database. It may be useful for characterization of the unknown sequences and identifying homologous sequences from the database. The users can also perform a customized search by choosing the type of matrix and an e-value cut-off.

#### (ii) Submission and update of HypoxiaDB

To make the database useful and to further improve it, it is essential to create an efficient and automatic data updating system. In this regard, we have prepared an online submission web page. The web page provides an intuitive interface to add the data. The data will be validated at an interval of 2–3 months and would be added automatically to the main HypoxiaDB database through a Perl script. If the users want to modify an already existing data within the database, they can write to the authors. The information would be validated and updated. The inputs from research community would continue to improve the quality and scope of the database. Our team is also continuously searching and adding new entries from published literature.

#### (iii) Feedback

An online feedback form is also provided to help improve HypoxiaDB and to meet the needs and requirements of the scientific community working in hypoxia and related disorders. Hypoxia research has been continuing to grow, and HypoxiaDB encourages users’ feedback, including error reports and feature requests, with the hope to make HypoxiaDB a comprehensive resource to facilitate hypoxia proteomic research, which may lead to some novel treatments.

#### (iv) Tutorial

For the convenience of the user, a detailed and self-explanatory tutorial is provided.

## Discussion

It has been reported that ∼1–1.5% of the genome is transcriptionally responsive to hypoxia; nevertheless, the response varies in different cell types in a time-dependent manner (35, 37). Calculating the chromosome-wise distribution pattern of HypoxiaDB proteins, it was found that maximum number of proteins are linked to chromosome number 1 (11%), followed by chromosome number 2 (8%) and 17 (6%). For the proteins located on sex chromosomes, 63 proteins were found to be located to chromosome X and only 3 proteins were found to be present on chromosome Y (Table 1). Earlier studies on chromosomal mapping of ischemia-hypoxia response genes have shown that high numbers of ischemia-hypoxia response genes were present on chromosomes 1, 2, 6, 7, 17 and 19 (38). Also, studies in mice have shown that hypoxic and hypercapnic breathing are genetically linked to mouse chromosomes 1 and 5 (39).

**Table 1.** Chromosome-wise distribution pattern of hypoxia-regulated proteins

Chromosome number	Number of proteins
1	251
2	177
3	134
4	85
5	106
6	117
7	129
8	94
9	81
10	93
11	127
12	122
13	27
14	86
15	60
16	93
17	135
18	37
19	133
20	59
21	28
22	49
X	63
Y	6

**Figure 4.**
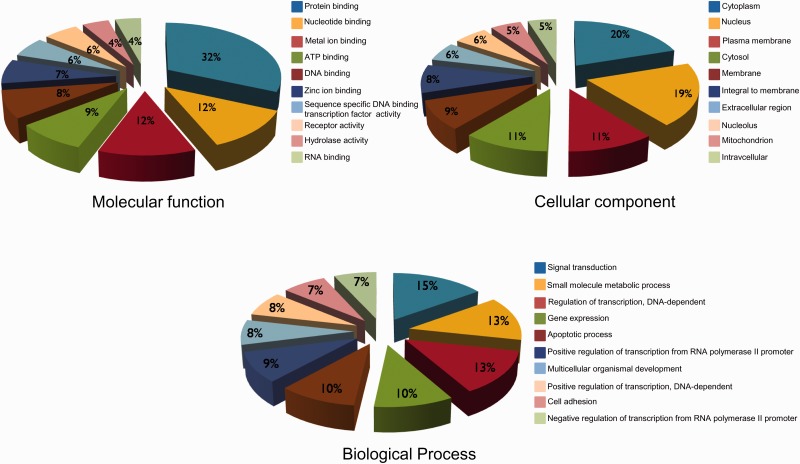
The summary of top 10 GO descriptions (based on molecular function, cellular component and biological processes) of hypoxia-regulated proteins in HypoxiaDB.

GO annotation have been used widely to characterize protein functions and to divulge trends in protein datasets; we have also classified hypoxia-regulated proteins according to their molecular function, cellular component and biological process. Assignment of molecular functions to the set of proteins revealed that the top five molecular function categories represented in the dataset are protein binding followed by nucleotide binding, metal ion binding, ATP binding and DNA binding (Figure 4). This suggests the importance of these proteins in complex protein interactions. Nucleotide binding proteins may represent some important transcription factors causing various cascading effects in the whole pathway. It was also observed that most of the proteins analyzed are localized in cytoplasm followed by nucleus. Their localization in the nucleus also supports the possibility of them being transcription factors (Figure 4). The biological processes in HypoxiaDB are enriched in signal transduction, small molecule metabolic process, regulation of transcription, gene expression and apoptotic process (Figure 4). This suggests that the proteins regulated by hypoxia may be involved in transition of cell progression to hypoxic conditions, which cause the change in gene and protein expressions, eventually leading to the death of the cells due to hypoxia. Many other research findings have also reported the phenomena of hypoxia-induced apoptosis (20, 34, 40, 41).

**Figure 5.**
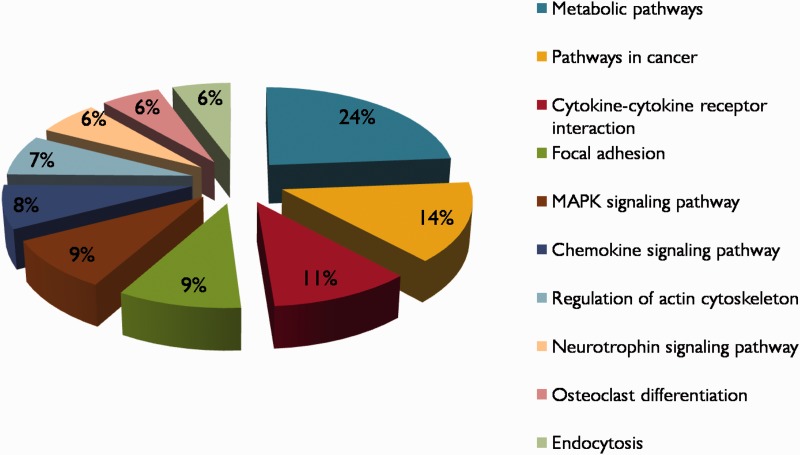
The top 10 KEGG pathway descriptions of hypoxia-regulated proteins in HypoxiaDB.

Hypoxia is a complex condition and there may be huge biological networks and pathways contributing to the pathogenesis of hypoxia. In this view, each protein in the database is linked with KEGG pathway and PPI information/networks. The KEGG pathways enriched in HypoxiaDB are metabolic pathways, pathways in cancer, cytokine–cytokine receptor interaction, focal adhesion and MAPK signaling pathways (Figure 5). Thus, to understand the molecular mechanisms in hypoxia, more attention should be paid to the analysis of these pathways, which contain many hypoxia-regulated proteins. Furthermore, it is also important to analyze proteins involved in multiple pathways, as they may possibly act as linkers among these pathways.

**Figure 6.**
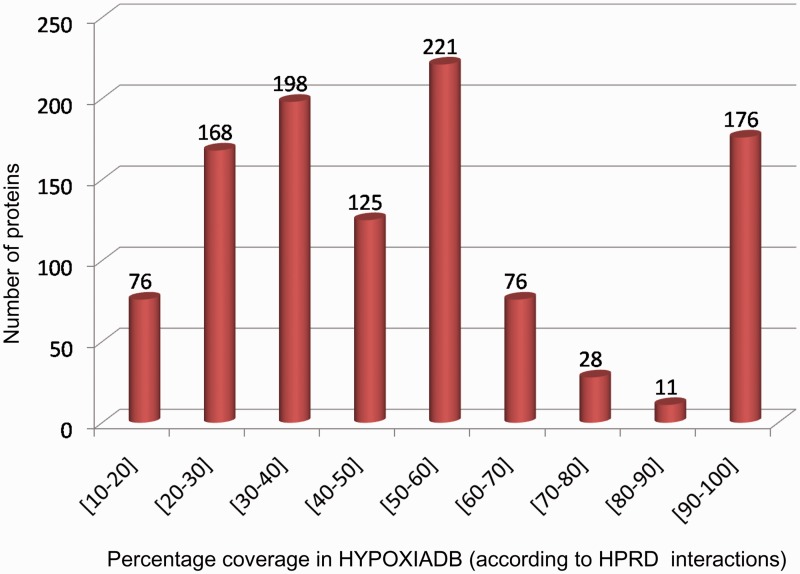
The coverage of database in terms of interacting partners within HypoxiaDB.

The PPI link allows the user to graphically view and study the interacting protein networks. Interestingly, the user can also identify the interacting partners within HypoxiaDB and elucidate major hypoxia-linked pathways/networks. Analyzing the interacting partners for each protein in HypoxiaDB, it was found that 215 proteins have their 75% interacting partners within HypoxiaDB itself (Figure 6). Notably, MAPK1 protein showed the highest interacting partners (99), and almost half of them were hypoxia-regulated proteins from HypoxiaDB. This proves a good coverage of HypoxiaDB and also helps to identify strong hypoxia-linked network complexities.

**Figure 7.**
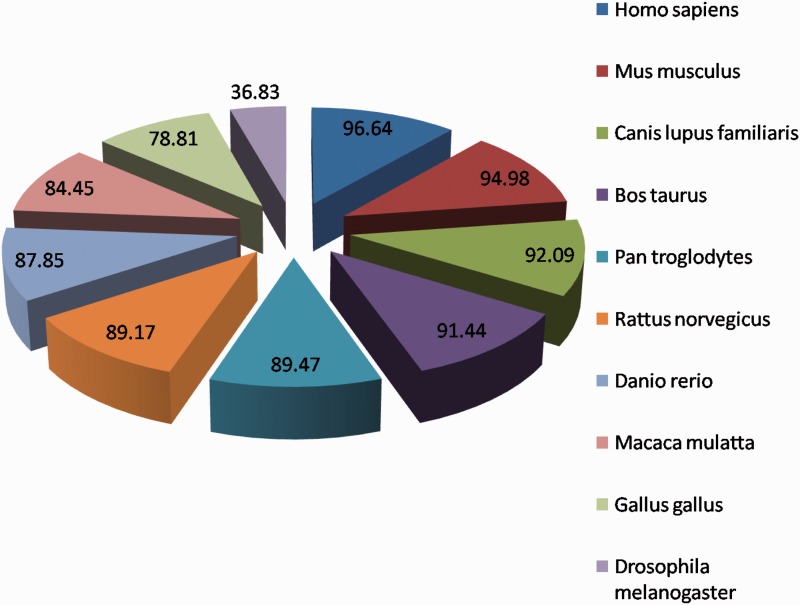
The top homologous species of proteins in HypoxiaDB.

The homology information helps user to identify conserved patterns present in the proteins during evolution and their ability to tolerate mutations in the sequence. The homologous protein analysis showed that most of the HypoxiaDB proteins (except 39) exhibit homology to other eukaryotic genomes; it suggests that hypoxia-regulated proteins have been conserved over the years in eukaryotic domain of life. The proteins exhibit homologous sequences in other genomes; the majority is conserved in *Mus musculus* (mouse) followed by *Canis lupus familiaris* (Dog)*, Bos taurus* (Cattle) and *Pan troglodytes* (Chimpanzee) (Figure 7), and only a small fraction is conserved in *Plasmodium falciparum.* This convergent evolution provides eukaryotes better ability to respond to low-oxygen conditions and offers them a competitive Darwinian advantage (35). The human hypoxia-regulated proteins have low homology in plants, as only a fraction of them are found to be conserved in *Arabidopsis thaliana* (2.9%) and *Oryza sativa* (2.2%). The presence of homologous proteins in various high complexity organisms implies that these proteins have evolved late along the evolutionary line. These proteins are probably responsible for varying oxygen adaptability and may help in distinguishing natural habitats of complex and simple eukaryotes. They may also help to distinguish between eukaryotic and prokaryotic mode of life. Many other research publications also highlight that prokaryotes and lower eukaryotes have simple mechanisms to respond to low-oxygen concentrations, whereas the mammalian cells have a complicated hypoxia response, which involves multiprotein complexes to regulate several transcription factors (42, 43). All these again emphasize that unicellular and multicellular organisms have evolved different mechanisms to maintain oxygen homeostatis under hypoxic stress.

We have compared HypoxiaDB with some of the popularly used generalized databases like AmiGO (22), GeneCards (23) and ArrayExpress/GenAtlas (24). AmiGO reported 17 proteins to be hypoxia proteins, which are only a fraction of the proteins collated in HypoxiaDB (Supplementary Figure S1A). GeneCards database reports 1538 proteins as hypoxia-related proteins. GeneCards search is a phrase-based search engine and looks for association between gene/protein and disease based on co-occurrence in abstracts. Both these databases do not provide tissue-specific expression profile of the protein under different levels and durations of hypoxia. ArrayExpress reports 18 757 proteins to be hypoxia-regulated from only four GEO IDs (corresponding to four publications). Of 18 757 proteins, 18 476 are reported from one GEO ID, i.e. E-GEOD-9649. Of these, only 1109 are significantly up/downregulated (based on *P* < 0.0000005). Besides, ArrayExpress would take data only from GEO dataset. There is a huge gap between published data and GEO datasets. Many experiments use Serial Analysis of Gene Expression (SAGE), Fluorescence In-Situ Hybridization (FISH) and western blots to identify the differentially expressed proteins; these are not deposited in the GEO database. HypoxiaDB currently contains 72 000 manually curated entries taken on 3500 differentially regulated hypoxia proteins extracted from 73 peer-reviewed publications selected from PubMed.

Using a specific hypoxia-regulated protein (namely PGK1), the results from the three web servers (ArrayExpress, GeneCards, AmiGo) were compared with that from HypoxiaDB (Supplementary Figure S1A–G). Searching AmiGO database for hypoxia genes/proteins retrieved 17 results (Supplementary Figure S1A). PGK1 was not listed in these results. Exploring the ‘term association’ tab on the PGK1 page in AmiGO database also did not report any association with hypoxia (Supplementary Figure S1B). ArrayExpress reports PGK1 being overexpressed in only two experiments (E-MEXP-445 and E-GEOD-9649) (Supplementary Figure S1C). In contrast, HypoxiaDB reports 15 association studies for PGK1 and hypoxia (Supplementary Figure S1F). The PGK1 page in the GeneCards database shows the expression profile of PGK1 in different tissue types (Supplementary Figure S1D). However, it does not provide the expression profile of the protein with respect to duration and level of hypoxia. HypoxiaDB uniquely provides the tissue-wise expression pattern of the proteins in different levels and durations of hypoxia (Supplementary Figure S1F). It additionally provides manually curated literature references to support the inclusion of the proteins in the database and establish the association with hypoxia. Furthermore, the database assimilates data on GO, KEGG pathway, PPIs, protein family, OMIM, PDB structures and homology to other sequenced genomes (Supplementary Figure S1G). HypoxiaDB allows researchers to search interested proteins that have been reported to be regulated in the hypoxic conditions, to compare their proteomic discovery with the previously published data and to relate the protein expression changes under various hypoxic conditions.

## Conclusion

With the considerable increase of hypoxia-regulated molecular data over the past few years generated by the use of various research techniques, including high-throughput transcript and proteomic analysis, there was a need to develop a database to facilitate hypoxia research at a molecular level. Yet, to date, to our knowledge, there is no resource available that provides detailed information about the proteins known to be associated with various hypoxic conditions. Therefore, we compiled the first hypoxia database ‘HypoxiaDB’, a comprehensive non-redundant catalog of proteins where manual curation along with the information from other resources has been integrated to provide a knowledgebase that will allow researchers and clinicians an overview of biology of the proteins involved in hypoxia and its related disorders. The data are presented in a systematic way, and apart from the search facility, many browsing options facilitate fast, efficient and user-friendly retrieval of information. HypoxiaDB serves as a ‘one-stop shop’ database, where information regarding a particular protein can be extracted from other databases and enriched with information from several additional analyses not obtainable from other repositories. It is also cross-referenced to external databases as leverage for harnessing new biological insights. To find homologous protein sequences from the database based on sequence similarity and for the characterization of orphan protein sequences, HypoxiaDB has a customized protein database that could be used with BLASTP, and this helps to provide one more layer of utility to this hypoxia database. Such layered curation and annotation of hypoxia-related proteins could be useful for better understanding of both the value of proteomics study in hypoxia and hypoxia-related research and the biological meaning of protein expression change under varying hypoxic conditions and tissue types.

We hope that HypoxiaDB would help in improving the existing knowledge about hypoxia and generation of some useful hypothesis and novel therapeutic strategies. This hypoxia-specific database would not only save time and effort of researchers but would also assist in deeper understanding of hypoxia biology.

## Future study

It is strongly proposed to update this database frequently and add new data from literature as well as other data analysis tools that will help improve the existing knowledge about hypoxia. More information like single nucleotide polymorphisms, transcription binding factors, motif analysis, etc will be added in the next version to make the database more useful for clinical as well as research purposes. Also, the present version has only human proteins; in the next version, we aim to include proteins of *Mus musculus* and *Rattus norvegicus*. We hope that this thorough and comprehensive database would be extensively used and sufficiently updated to enable efficient research in the field of hypoxia.

## Availability

HypoxiaDB is freely available at http://www.hypoxiadb.com.

## Supplementary Data

Supplementary data are available at *Database* Online.

Supplementary Data
