# Supplementary material for: HypoxiaDB: a database of hypoxia-regulated proteins
Source: Database (Oxford). 2013 Oct 30;2013:bat074. doi: 10.1093/database/bat074 (PMC3813937; doi:10.1093/database/bat074)
Supplement: Supplementary Data [file supp_2013_bat074_index.html]

Supplementary Data 

# HypoxiaDB: a database of hypoxia-regulated proteins

## Supplementary Data

files

**Files in this Data Supplement:**

- Supplementary Data - pptx file
