# Supplementary material for: HypoxiaDB: a database of hypoxia-regulated proteins
Source: Database (Oxford). 2013 Oct 30;2013:bat074. doi: 10.1093/database/bat074 (PMC3813937; doi:10.1093/database/bat074)
Supplement: Supplementary Data [file supp_bat074_Supplementary_figure_1.pptx]

## Slide 1
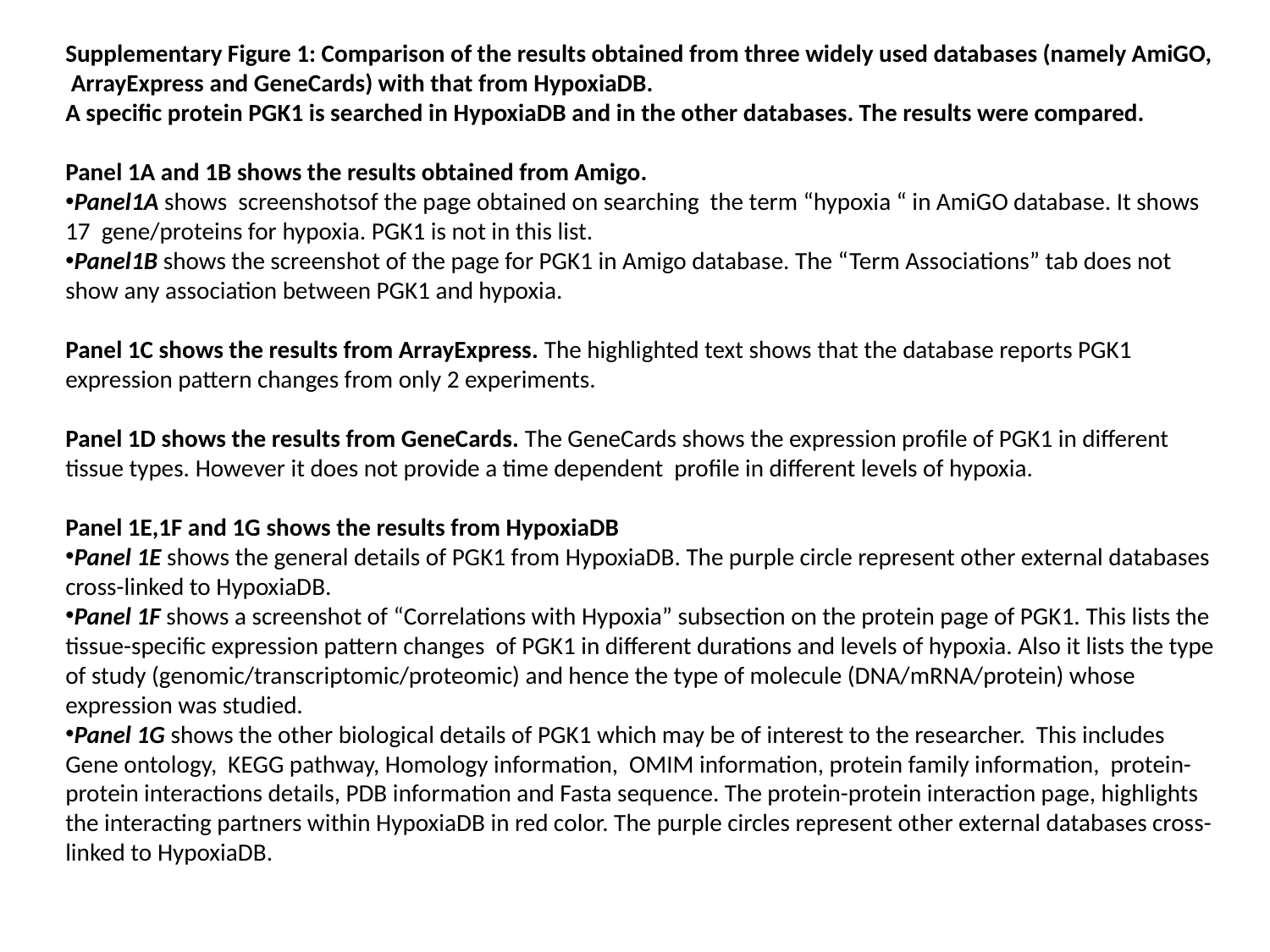

Supplementary Figure 1: Comparison of the results obtained from three widely used databases (namely AmiGO, ArrayExpress and GeneCards) with that from HypoxiaDB.
A specific protein PGK1 is searched in HypoxiaDB and in the other databases. The results were compared.
Panel 1A and 1B shows the results obtained from Amigo.
Panel1A shows screenshotsof the page obtained on searching the term “hypoxia “ in AmiGO database. It shows 17 gene/proteins for hypoxia. PGK1 is not in this list.
Panel1B shows the screenshot of the page for PGK1 in Amigo database. The “Term Associations” tab does not show any association between PGK1 and hypoxia.
Panel 1C shows the results from ArrayExpress. The highlighted text shows that the database reports PGK1 expression pattern changes from only 2 experiments.
Panel 1D shows the results from GeneCards. The GeneCards shows the expression profile of PGK1 in different tissue types. However it does not provide a time dependent profile in different levels of hypoxia.
Panel 1E,1F and 1G shows the results from HypoxiaDB
Panel 1E shows the general details of PGK1 from HypoxiaDB. The purple circle represent other external databases cross-linked to HypoxiaDB.
Panel 1F shows a screenshot of “Correlations with Hypoxia” subsection on the protein page of PGK1. This lists the tissue-specific expression pattern changes of PGK1 in different durations and levels of hypoxia. Also it lists the type of study (genomic/transcriptomic/proteomic) and hence the type of molecule (DNA/mRNA/protein) whose expression was studied.
Panel 1G shows the other biological details of PGK1 which may be of interest to the researcher. This includes Gene ontology, KEGG pathway, Homology information, OMIM information, protein family information, protein-protein interactions details, PDB information and Fasta sequence. The protein-protein interaction page, highlights the interacting partners within HypoxiaDB in red color. The purple circles represent other external databases cross-linked to HypoxiaDB.

## Slide 2
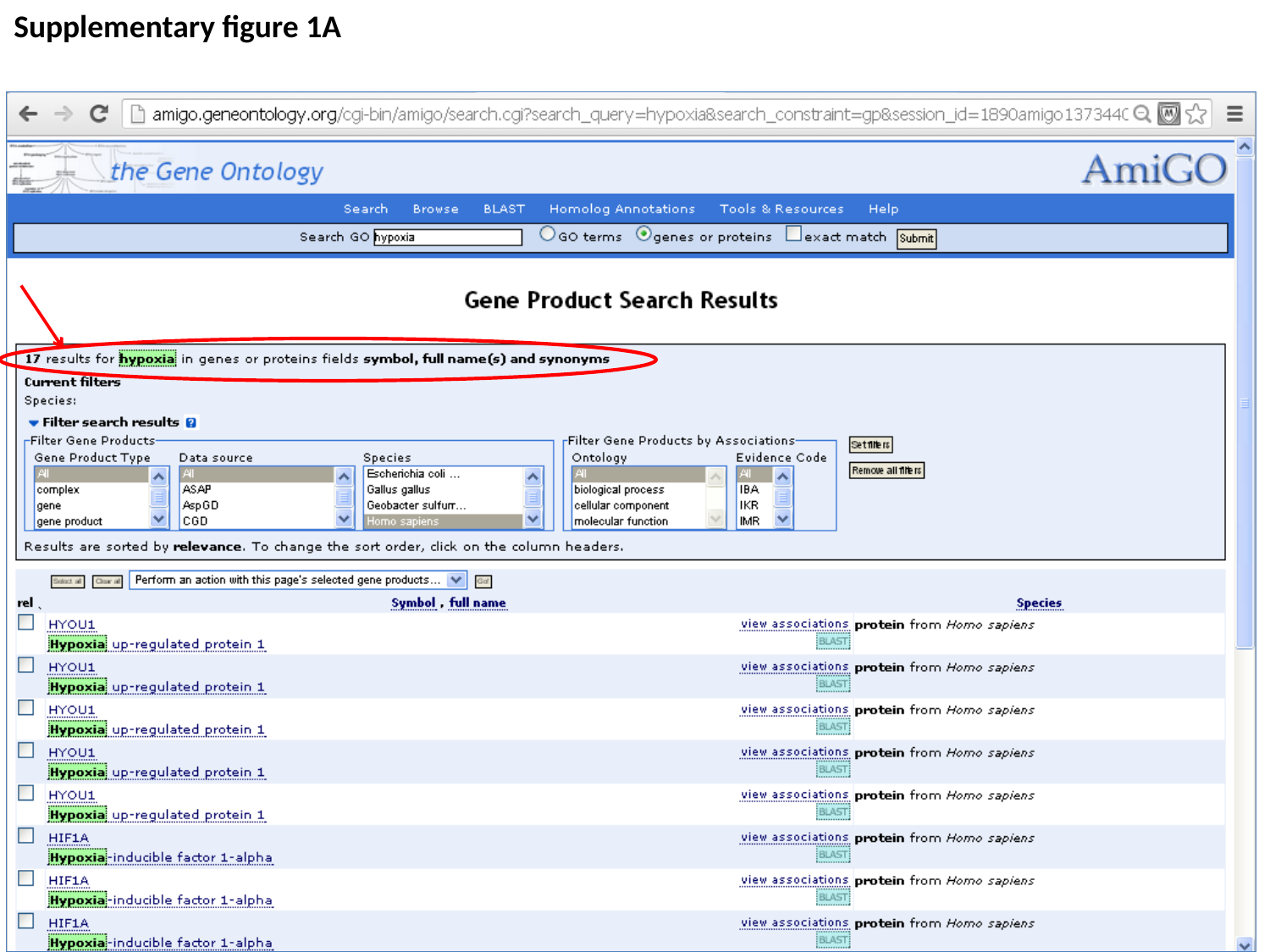

Supplementary figure 1A

## Slide 3
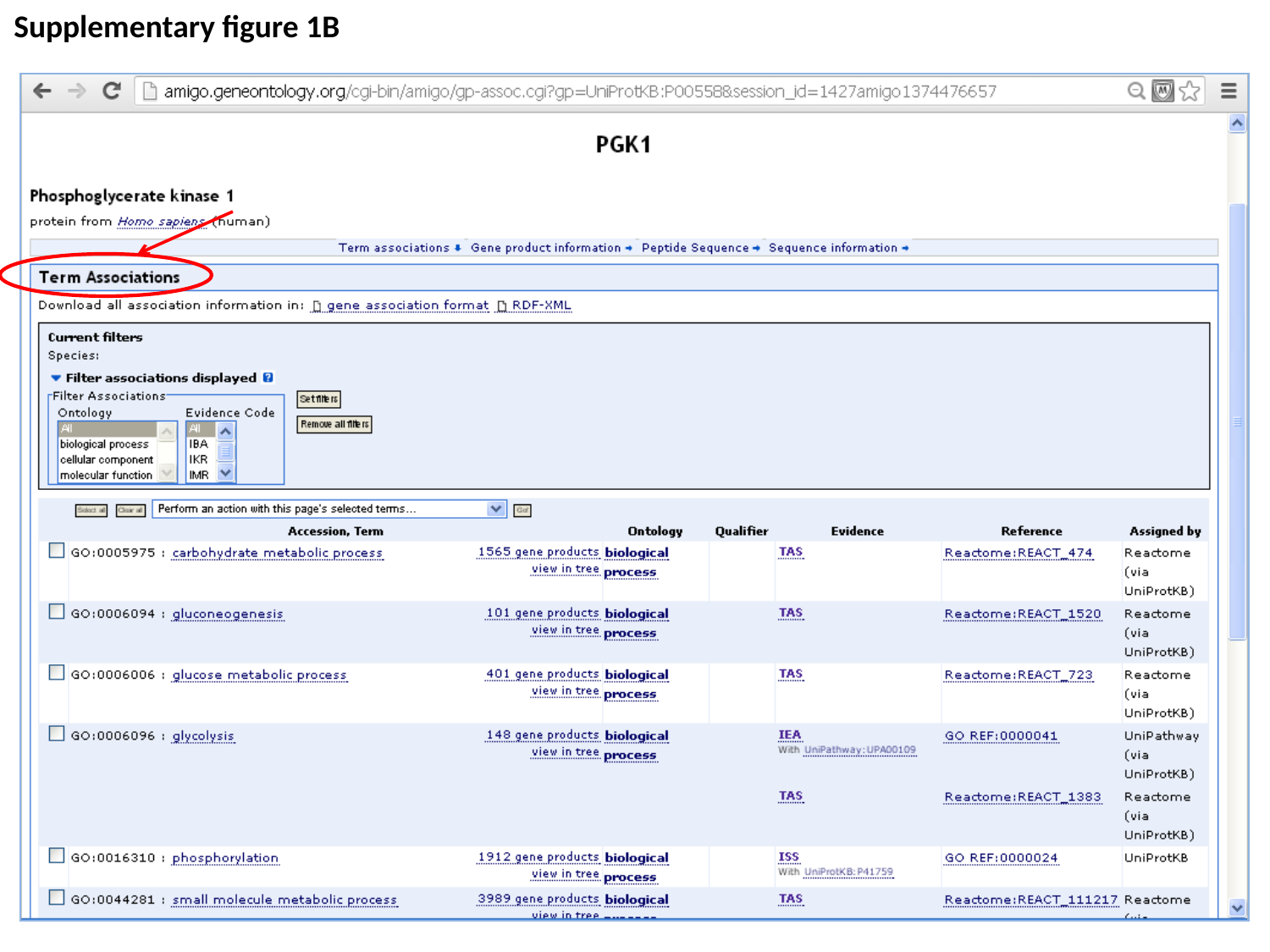

Supplementary figure 1B

## Slide 4
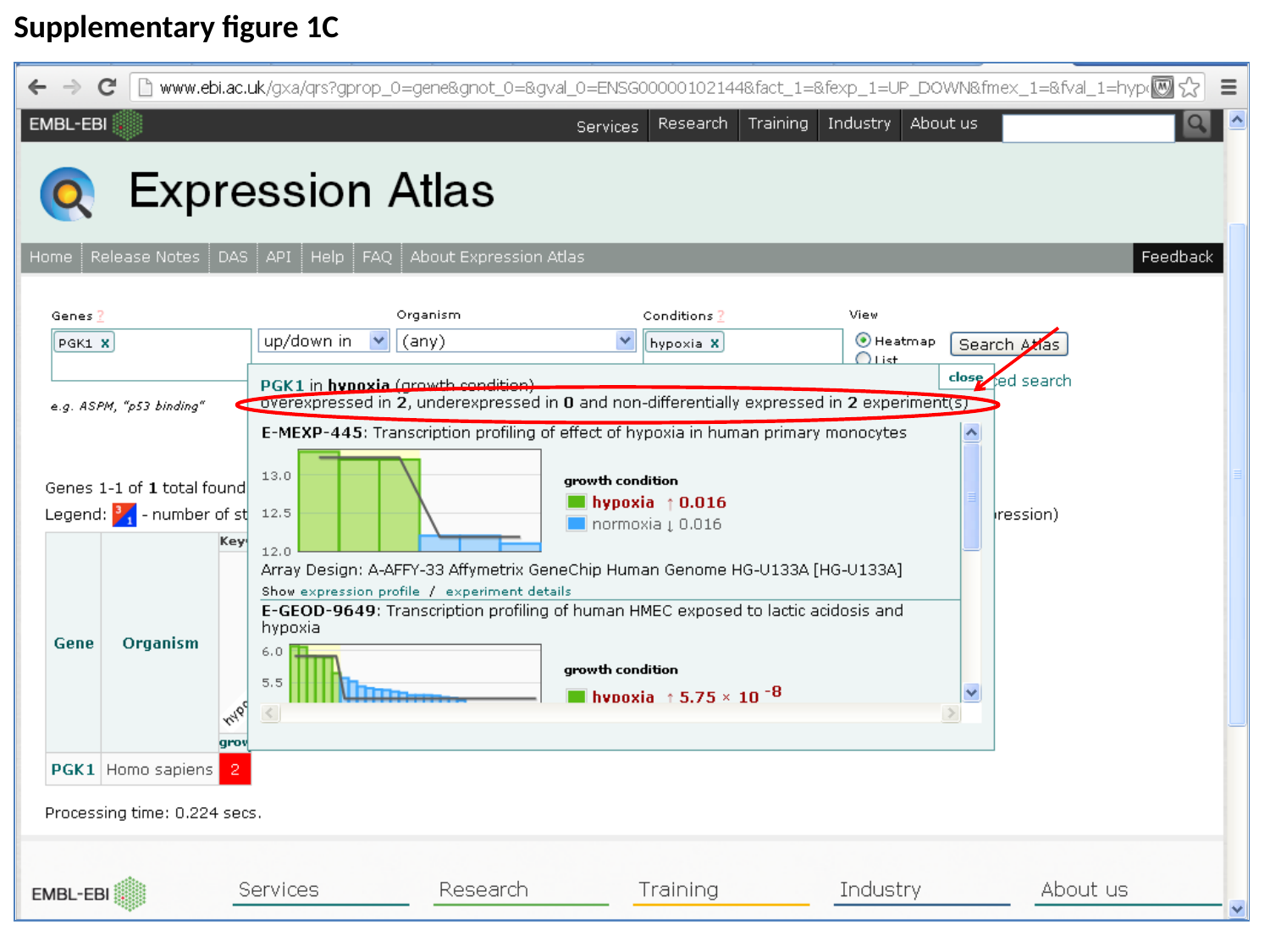

Supplementary figure 1C

## Slide 5
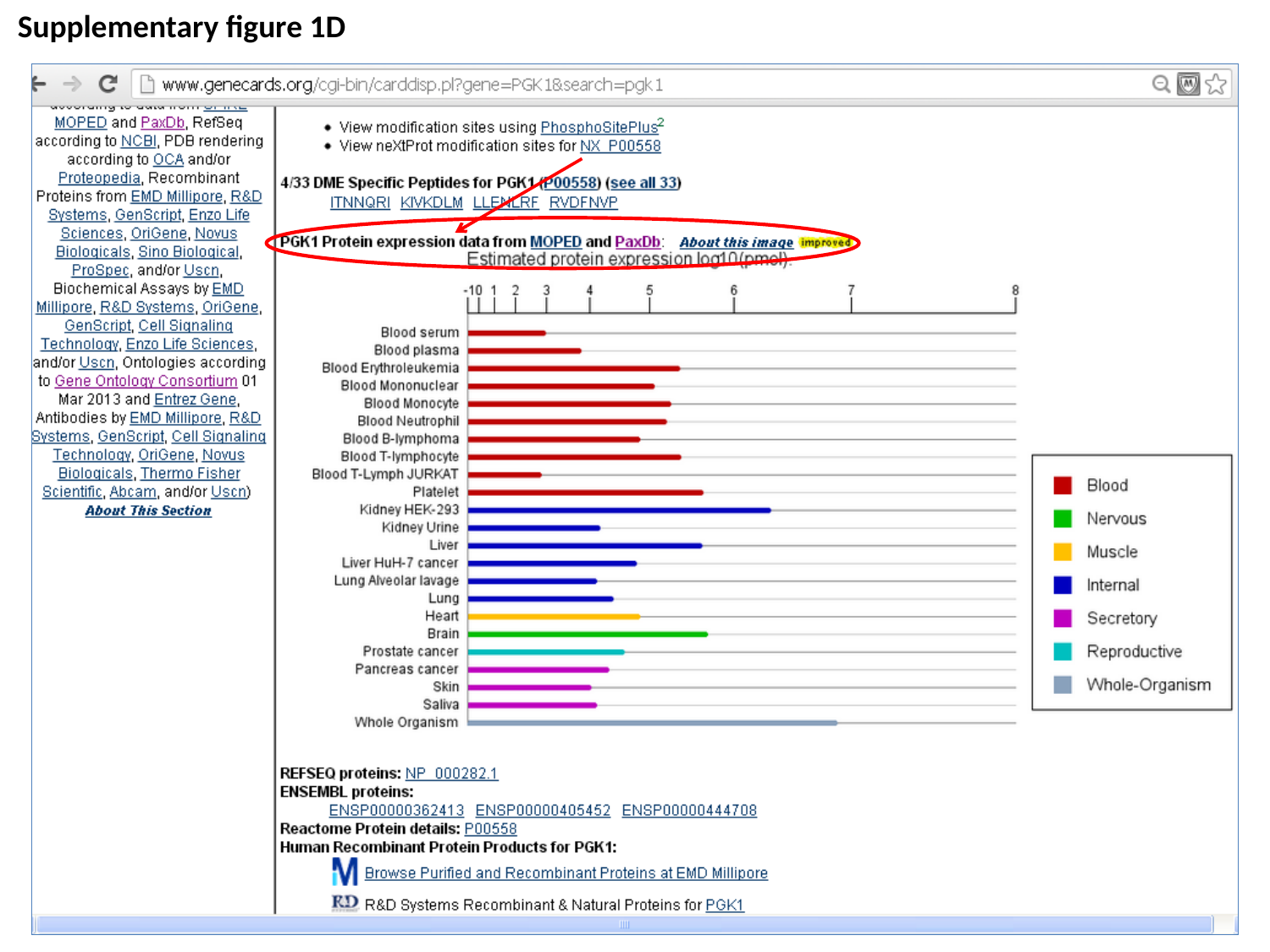

Supplementary figure 1D

## Slide 6
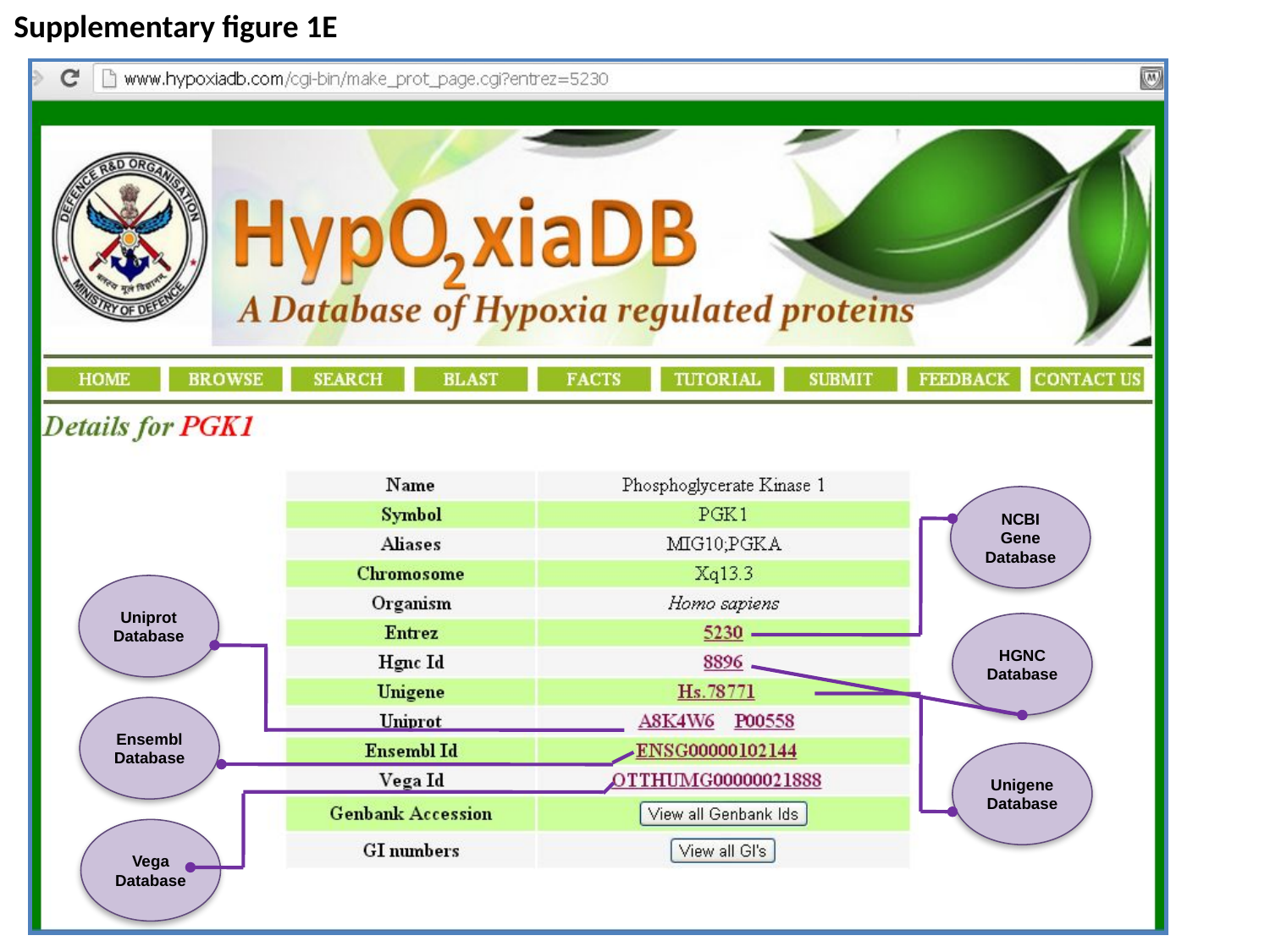

Supplementary figure 1E
NCBI Gene Database
Uniprot
Database
HGNC
Database
EnsemblDatabase
UnigeneDatabase
Vega
Database

## Slide 7
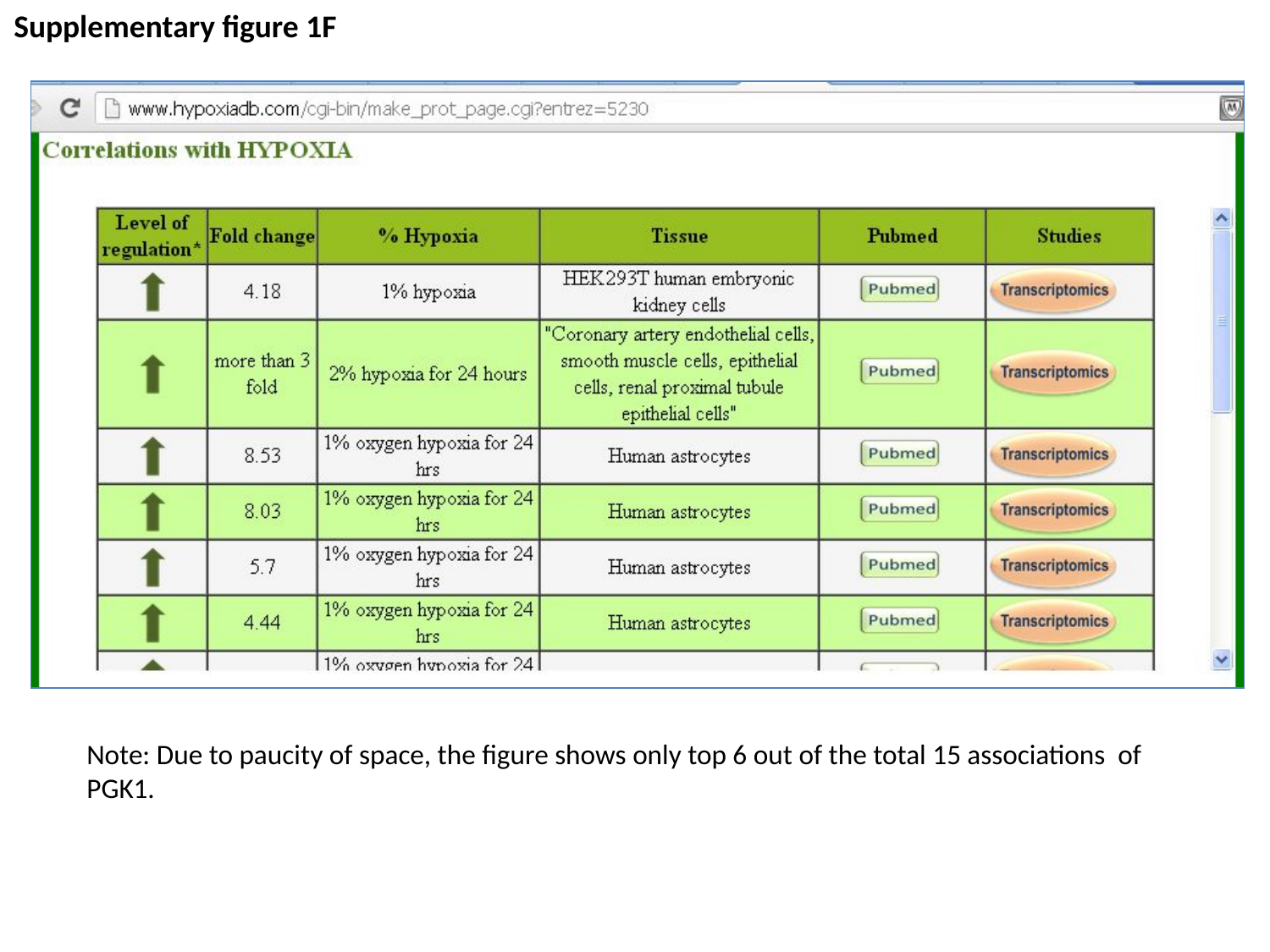

Supplementary figure 1F
Note: Due to paucity of space, the figure shows only top 6 out of the total 15 associations of PGK1.

## Slide 8
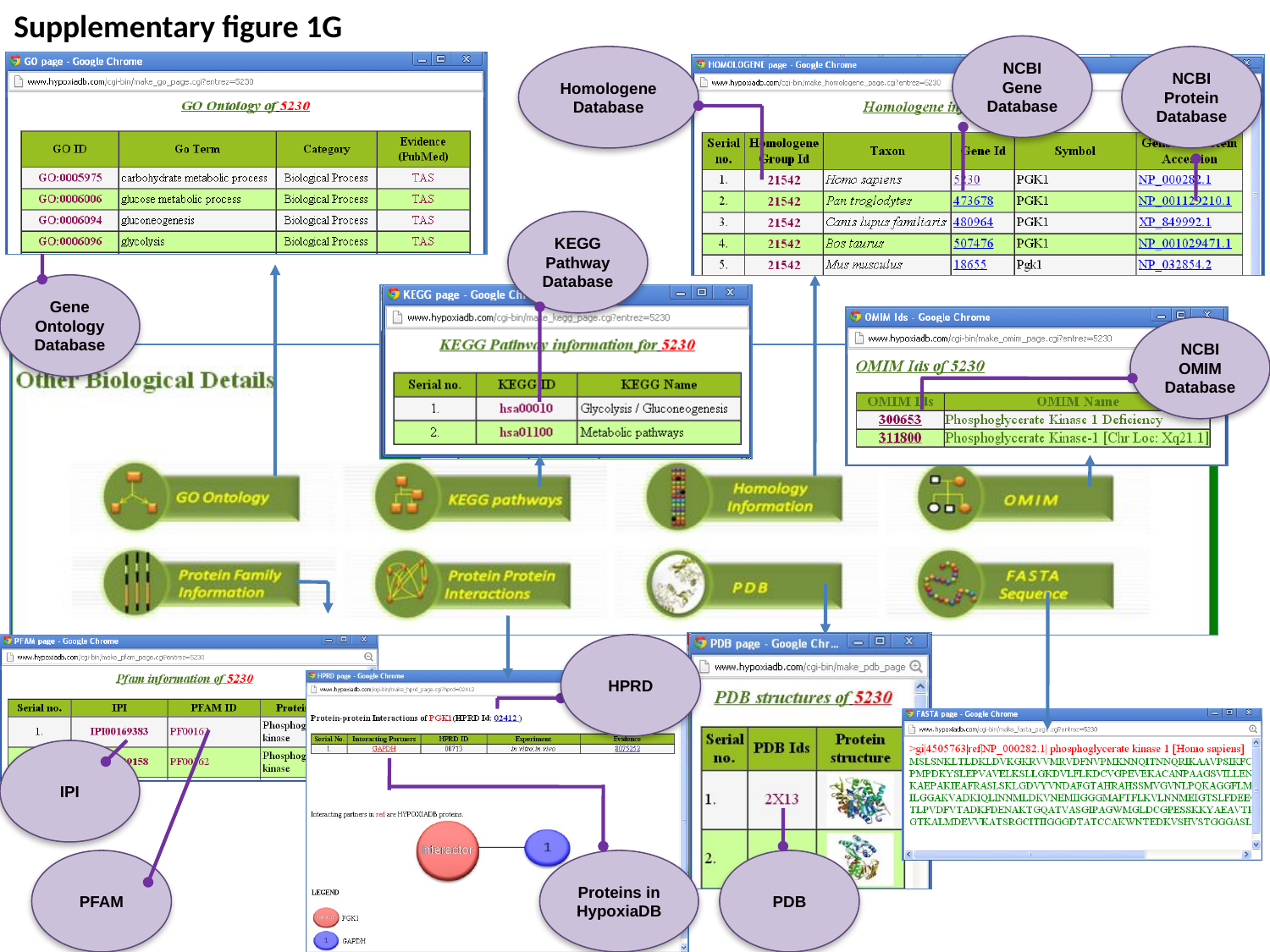

Supplementary figure 1G
NCBI Gene Database
Homologene Database
NCBI Protein Database
KEGG Pathway Database
Gene Ontology Database
NCBI OMIM Database
HPRD
IPI
PFAM
Proteins in HypoxiaDB
PDB
